# Green and Highly-Efficient Microwave Synthesis Route for Sulfur/Carbon Composite for Li-S Battery

**DOI:** 10.3390/ijms23010039

**Published:** 2021-12-21

**Authors:** Chun-Han Hsu, Cheng-Han Chung, Tzu-Hsien Hsieh, Hong-Ping Lin

**Affiliations:** 1 General Education Center, National Tainan Junior College of Nursing, Tainan 700, Taiwan; 2 Department of Chemistry, National Cheng Kung University, Tainan 70101, Taiwan; e84011184@gmail.com; 3 Green Technology Research Institute, CPC Corporation, Kaohsiung 81126, Taiwan; 295931@cpc.com.tw

**Keywords:** porous carbon, template, biomass pyrolysis oil, battery

## Abstract

Multiporous carbons (MPCs) are prepared using ZnO as a hard template and biomass pyrolysis oil as the carbon source. It is shown that the surface area, pore volume, and mesopore/micropore ratio of the as-prepared MPCs can be easily controlled by adjusting the ZnO/oil ratio. Sulfur/MPC (S/MPC) composite is prepared by blending sulfur powder with the as-prepared MPCs followed by microwave heating at three different powers (100 W/200 W/300 W) for 60 s. The unique micro/mesostructure characteristics of the resulting porous carbons not only endow the S/MPC composite with sufficient available space for sulfur storage, but also provide favorable and efficient channels for Li-ions/electrons transportation. When applied as the electrode material in a lithium-ion battery (LIB), the S/MPC composite shows a reversible capacity (about 500 mAh g^−1^) and a high columbic efficiency (>95%) after 70 cycles. Overall, the method proposed in this study provides a simple and green approach for the rapid production of MPCs and S/MPC composite for high-performance LIBs.

## 1. Introduction

Lithium-ion batteries (LIBs) are regarded as an attractive power source for a wide variety of applications nowadays, including energy storage systems and electric vehicles [1,2,3,4]. However, despite extensive research, the energy density of LIBs is still limited by the low capacity of current cathode materials. For example, LiCoO_2_, LiFePO_4_, and LiNi_x_Co_y_Al_z_O yield specific capacity ranges of just 150 to 250 mAh g^−1^ [5,6]. Sulfur has many advantages as a potential cathode material, including a high theoretical specific capacity of 1675 mAh g^−1^, natural abundance, low cost, and environmental friendliness [7,8,9]. However, the low electrical conductivity of sulfur (~10^−30^ S cm^−1^), together with the tendency of the polysulfides to dissolve into electrolytes and a significant volume expansion effect during cycling, limits its practical application in rechargeable LIB [9,10,11]. Ideally, the sulfur should be locked and bound in certain carbon structures in order to prevent it from making direct contact with the electrolyte [12,13]. Under such conditions, the redox reaction between the sulfur and Li ions occurs mostly in the carbon skeleton, thereby improving the electron conductivity and enhancing the Li-ion transfer. Accordingly, the literature contains many nanostructured materials consisting of sulfur and various types of carbon for enhancing the cycling stability of sulfur-based electrodes [13,14,15]. However, the preparation of sulfur/carbon (S/C) composite via traditional thermal treatment routes is time consuming and expensive. Thus, more efficient methods for the mass production of sulfur-based carbon composites for LIB applications are urgently required.

Porous carbons are an attractive material for many applications, including electrode for supercapacitor, adsorbent, and catalyst supporter [16,17,18]. Among the many porous carbon materials available, multiporous carbons (MPCs) with three-dimensional mesoporous/microporous structures have attracted particular attention due to their high specific surface area, good chemical inertness, high electrical conductivity, and tunable porosity [19,20]. MPCs are used for many applications nowadays, including as catalyst supports, and electrode materials for fuel cells, LIBs, and supercapacitors [21,22]. Porous carbons are usually synthesized using siliceous templates. However, the synthesis process is expensive, complicated, and time-consuming, and is thus impractical for large-scale production [23,24]. By contrast, non-siliceous templates, such as ZnO and CaCO_3_, are chemically inert and more environmentally friendly [25,26]. Consequently, non-siliceous templates, which can be removed using a suitable acid solution, are regarded as a more promising approach for the synthesis of porous carbons.

Traditionally, porous carbons have always been prepared using resin as a carbon source. However, while many different porous carbons have been successfully synthesized in this way, they have a high cost and a low conductivity [27,28]. Biomass pyrolysis oil is cheap and abundant and has a relatively high conductivity due to its rich benzene rings [29]. Furthermore, the reduction of biomass pyrolysis oil to high-value porous carbons is an environmentally-friendly process. As a result, biomass utilization has emerged as an important carbon reduction technology in recent years. The CPC Corporation in Taiwan, for example, has developed a proprietary biomass pyrolysis technology with good stability for feedstock loads as high as 400 kg [30]. The present study proposes a novel synthesis route for the production of MPCs using ZnO as a hard template and biomass pyrolysis oil as the carbon source. Notably, the ZnO templates can be removed using a simple hydrochloric acid solution, thereby overcoming the limitation of conventional silica templating approaches, wherein highly corrosive hydrofluoric acid is required to remove the templates. It is shown that the mesoporous surface area of the MPCs can be tuned within the range of approximately 1131 to 1678 m^2^ g^−1^ simply by increasing the ZnO/oil weight ratio from 2 to 5. Furthermore, the MPCs have a high specific surface area of approximately 1280–1770 m^2^ g^−1^ without the need for any additional activator agent (e.g., KOH). Carbon materials are, in general, very good absorbents of microwaves [31]. The microwaves are transformed into heat inside the carbon particles by dipole rotation, and heat the carbon directly [32,33]. Thus, in the present study, the as-prepared MPCs are blended with sulfur powder, and the mixture is then microwaved to produce S/MPC composite material. The synthesized S/MPC composite is used as the electrode material in a LIB and is shown to have both a large reversible capacity (about 500 mAh g^−1^) and a high columbic efficiency (>95%) after 70 cycles.

## 2. Results and Discussion

### 2.1. Effect of ZnO/Oil Weight Ratio on Textile Properties of MPCs

**Figure 1:**
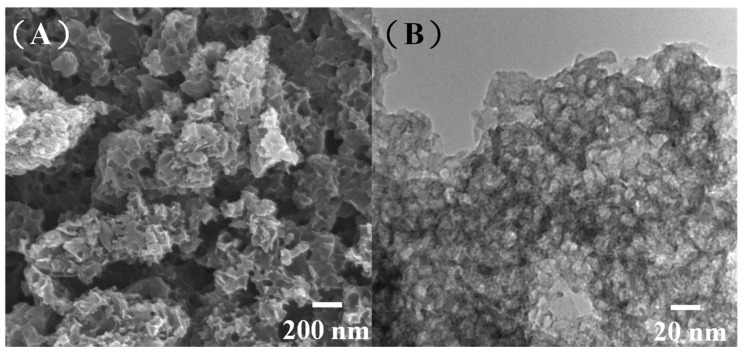
(**A**) Scanning electron microscopy (SEM) and (**B**) transmission electron microscopy (TEM) images of multiporous carbons (MPCs) with ZnO/oil ratio of z = 3.

The present group recently proposed a method for the solvent-free synthesis of MPCs with tailored porosity using ZnO nanoparticles as a hard template and pitch as the carbon source [34]. Herein, the pitch was replaced with biomass pyrolysis oil, and the effect of the ZnO/oil ratio (z) on the textile properties of the resulting MPCs was examined. Figure 1A,B present scanning electron microscopy (SEM) and transmission electron microscopy (TEM) images of the MPCs produced using a ZnO/oil ratio of z = 3. The ZnO nanoparticles used in the synthesis process had a spherical form with a size of approximately 20 nm (see Figure S1). After removing the ZnO templates by HCl etching, the pore size of the porous carbon was close to that of the original ZnO particles (see Figure 1A), which indicates that the pores within the MPC were formed via the integral casting of the biomass pyrolysis oil on the ZnO templates. The TEM image in Figure 1B confirms that the MPC has a highly porous structure with thin and unbroken carbon walls.

**Figure 2:**
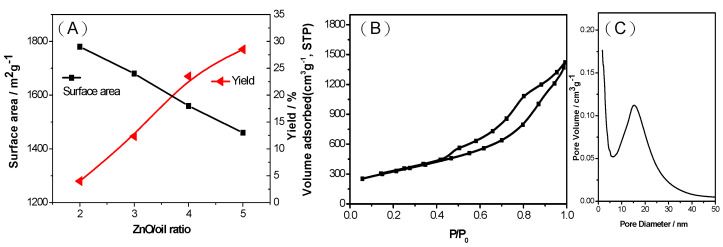
(**A**) Surface area and carbon yield of MPCs with ZnO/oil ratio of z = 2 to 5, (**B**) nitrogen adsorption-desorption isotherms, and (**C**) pore size distribution curves for MPC with ZnO/oil ratio of z = 3.

The textural properties of carbon materials have a critical effect on their performance [35]. In the present study, the surface area and pore volume of the MPCs were controlled simply by adjusting the ZnO/oil ratio, as shown in Figure 2A. The isotherm profiles shown in Figure 2B for the MPC with a ZnO/oil ratio of z = 3 exhibit well-defined capillary condensation steps, which are typical of a type IV isotherm. It is hence inferred that the MPC has a mesoporous structure with a pore size generally larger than 2.0 nm. This finding is confirmed by the pore size distribution curves in Figure 2C, which show that the MPCs consist mainly of large mesopores.

As shown in Table 1, the MPCs synthesized with ZnO/oil ratios of 2, 3, 4 and 5, respectively, have BET specific surface areas of 1280, 1447, 1670 and 1770 m^2^g^−1^. Furthermore, the mesoporosity of the MPCs increases sharply from 1131 to 1678 m^2^ g^−1^ as the ZnO/oil ratio increases from 2 to 5. Interestingly, the results show that the MPCs have a high meso-porosity and specific surface area without the addition of any activating agent, such as KOH. It is speculated that this may result from the liquid nature of the biomass pyrolysis oil carbon source, which allows it to mix thoroughly with the ZnO templates and uniformly coat them, thereby resulting in a strong reaction effect under a carbonization temperature of 900 °C. The textural parameters of the resulted porous carbon prepared at different weight ratios of ZnO to Oil are listed in Table 1. However, while an increasing ZnO/oil ratio is beneficial in increasing the specific surface area and mesoporosity of the MPCs, it also results in a lower carbon yield (see right-hand column in Table 1), and hence limits its application for downstream processing. Thus, in the sulfur loading and LIB trials conducted in the remainder of this study, the MPC ZnO/oil ratio was maintained at a constant z = 3, corresponding to a BET surface area of 1447 m^2^g^−1^ and a carbon yield of 24%.

**Figure 3:**
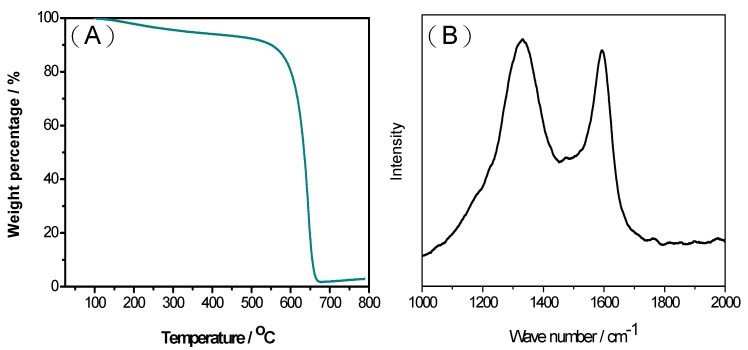
(**A**) Thermogravimetric analysis (TGA) curve and (**B**) Raman spectra of MPCs with ZnO/oil ratio of z = 3.

One of the key advantages of the MPC synthesis route proposed in this study is the easy removal of the ZnO templates using hydrochloric acid. As shown in Figure 3A, the residual ZnO following HCl etching was just ~2%. Moreover, the MPC material shows a high thermal stability at decomposition temperatures greater than 600 °C. In general, the results confirm the feasibility for synthesizing porous carbons using ZnO as the hard template rather than the more traditional SiO_2_ templates. The carbon graphitic structure and crystallinity of the MPCs with a ZnO/oil ratio of z = 3 were further investigated using Raman spectroscopy, as shown in Figure 3B. Carbon materials exhibit two main characteristic absorption bands, namely a G-band (~1580 cm^−1^) corresponding to the in-plane displacement of the carbon atoms in the hexagonal sheets, and the D-band (~1350 cm^−1^) associated with the disordering of the structure [36]. In general, the extent of the defects in carbon materials can be quantified by the intensity ratio of the D to G bands (i.e., I_D_/I_G_) [37]. For the present MPC material, the I_D_/I_G_ ratio is equal to just 0.91. This low value indicates that the structural damage of the carbon layers is caused by an increased porosity of the carbon. It is noted that this assertion is consistent with the TEM results shown in Figure 1B.

### 2.2. Structural Characterization of S/MPC and Application to Li-S Batteries

**Figure 4:**
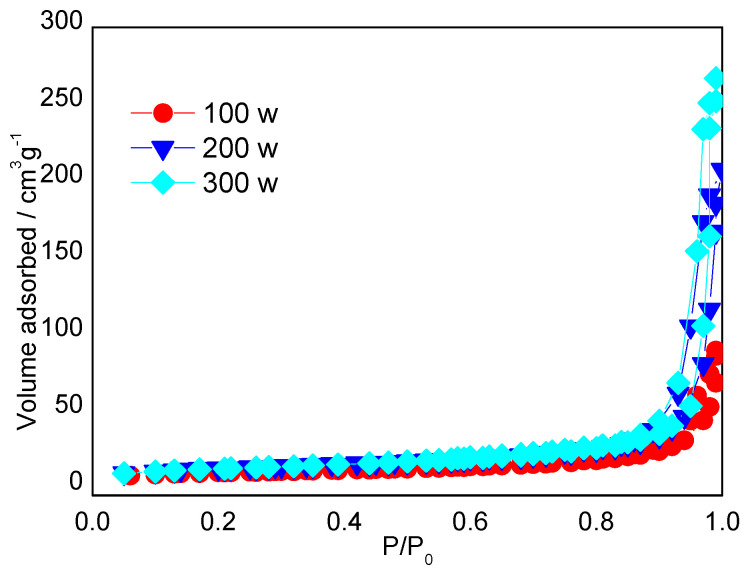
Nitrogen adsorption-desorption isotherms of the MPCs prepared with different microwave powers.

**Figure 5:**
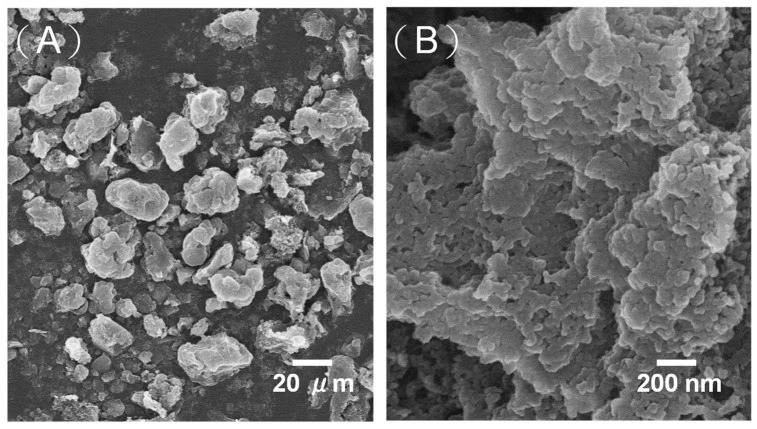
(**A**,**B**) Scanning electron microscopy (SEM) images of S/MPC sample prepared with microwave power of 100 W.

**Figure 6:**
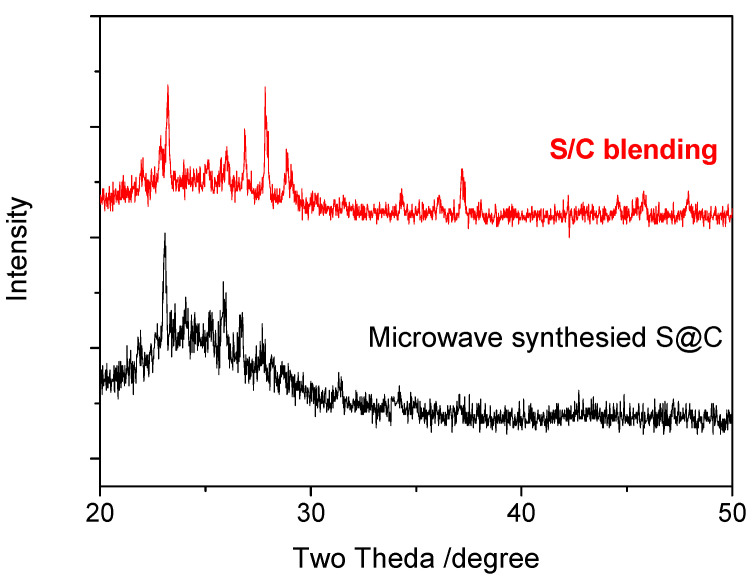
X-ray diffraction (XRD) patterns of S/MPC samples prepared by direct blending and microwave synthesis with power of 100 W.

MPCs enhance the electronic conductivity and wettability of the interface between the electrolyte and the electrode, and therefore represent an ideal electrode material for LIBs [22]. In the present study, S/MPCs were synthesized by blending the MPCs prepared above (with a ZnO/oil ratio of z = 3) with sulfur powder and then performing microwave treatment for 60 s. Figure 4 shows the N_2_ adsorption-desorption isotherms of the S/MPC materials obtained using microwave powers of 100 W, 200 W and 300 W, respectively. Unlike the raw MPC (see Figure 2B), the isotherms of the S/MPC materials do not exhibit well-defined capillary condensation steps (i.e., type IV isotherms), indicating that the pores are filled with sulfur. Moreover, the specific surface areas of the samples processed using powers of 100 W, 200 W and 300 W are just 32.5, 41.9 and 42.7 m^2^g^−1^, respectively, while the corresponding pore volumes are 0.13, 0.26 and 0.39 cm^3^ g^−1^ (see Table 2). The results presented in Table 2 show that the higher surface area and pore volume of the S/MPCs processed at higher powers result in a lower sulfur content. The SEM images presented in Figure 5 confirm that the S/MPC prepared with a power of 100 W has a non-porous surface, and is hence very different from that of the raw MPC sample shown in Figure 1A. Figure 6 shows the X-ray diffraction (XRD) patterns of the S/MPC mixture before and after microwave treatment with a power of 100 W. Note that the theoretical sulfur load is 80 wt.% in both cases. The original S/MPC product has an amorphous phase with a broad peak at around 24° corresponding to the (002) diffraction peak of carbon. In addition, the peak intensity of Sulfur in S/MPC sample is lower than that in the blended sample, which indicates that it has lower crystallinity. Overall, the results show that the microwave treatment process provides a simple yet effective means of filling the sulfur into the pores of the MPC material. The unique micro/mesostructure characteristics of the resulting porous carbons not only endow the S/MPC composite with sufficient available space for sulfur storage, but also provide favorable and efficient channels for Li-ions/electrons transportation.

**Figure 7:**
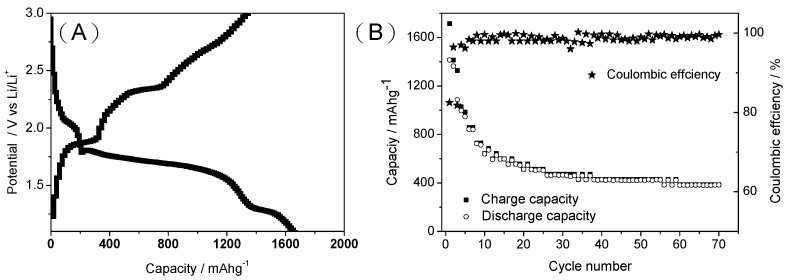
(**A**) First charge–discharge curve and (**B**) capacity and coulombic efficiency vs. cycle plot of S/MPC electrode.

Figure 7A shows the first charge–discharge curves of the S/MPC composite material when assembled as the electrode material in a LIB. The charge and discharge capacities at 200 mA g^−1^ are seen to be ~1600 and ~1400 mAh g^−1^, respectively, corresponding to a high irreversibly capacity of 20%. Moreover, as shown in Figure 7B, the S/MPC material has a high cycle stability with a capacity retention after 70 cycles as high as 90% of 20th cycles, and a columbic efficiency of up to 95% after the 5th cycle. By comparison, a pure S electrode has a short life time of less than 10 cycles, as shown in Figure S2 in the Supplementary Materials due to the dissolution of the polysulfide in the electrolyte during the charge/discharge procedure. Furthermore, the electrochemical impedance spectra (Figure S3) shows that the S/MPC electrode exhibited a lower charge-transfer resistances than sulfur electrode. In other words, the results confirm the effectiveness of the MPC network in preventing the dissolution of the polysulfide.

## 3. Experimental Section

### 3.1. Synthesis of MPCs

The MPCs were prepared using biomass pyrolysis oil (CPC Company, Taiwan) and ZnO (20-nm particles, Demonchem, Nantou, Taiwan) as the carbon source and hard template/activating agent, respectively. In a typical synthesis process, biomass pyrolysis oil and ZnO were ground into a homogeneous mixture with a mechanical blender without solvent. MPCs were prepared with four different ZnO/oil weight ratios, i.e., z = 2, 3, 4 and 5 (z = x/y, where x is the weight of ZnO and y is the weight of biomass pyrolysis oil). In each case, the powder was sealed in a stainless-steel vessel and heated at a rate of 8 °C min^−1^ to the desired carbonization temperature of 900 °C. The powder was maintained at this temperature for 2 h and was then allowed to cool naturally in the furnace to room temperature. The sample was washed with water and soaked in an appropriate amount of 3.0 M HCl aqueous solution to reach pH < 1.0) under stirring for 2 h to remove the alkaline oxide and ZnO template. Finally, the sample was filtered, washed with water and dried at 100 °C to obtain the desired MPC.

### 3.2. Preparation of S/MPC Composites

0.10 g MPC and 0.40 g sulfur powder (99%, Merck, Taipei, Taiwan) were ground into a homogeneous mixture with a mechanical blender without solvent. The mixture was transferred to a one end closed quartz tube and placed at the center of a microwave furnace (CYF-1kW, CHIN YING FA Mechanical Ind. Co., Ltd., Tainan, Taiwan). The samples were subjected to microwave heating at three different powers (100 W/200 W/300 W) for 60 s. All of the synthesis experiments were performed in a N_2_ atmosphere with a constant N_2_ flow rate of 100 cc min^−1^.

### 3.3. Characterization

The morphologies of the MPCs and S/MPCs were examined using a JEOL JEM6700 field emission scanning electron microscope operating at 10 kV. TEM observations were additionally performed using a Hitachi H-7500 microscope operating at 120 kV. The specific surface areas of the porous carbons were determined through the Brunauer–Emmett–Teller (BET) method on a Micromeritics Tristars 3020 instrument. In addition, the pore size distributions were analyzed by the BJH (Barrett-Joyner-Halenda) method with the Halsey equation for multilayer thickness. The carbon to inorganic template ratio of the particles was characterized in air using a TGA system (TA Instruments Q50, New Castle, DE, USA) with a ramp rate of 20 °C min^−1^ and a heating range of 100 to 800 °C. The structures of the various carbon samples were determined using a micro Raman spectrometer (Renishaw) fitted with a He-Ne laser source with a wavelength of 633 nm. Finally, XRD experiments were conducted with a scanning range of 2θ = 10–60° on a Philips X-pert diffractometer with CuK*α* radiation with a wavelength of *λ* = 1.540 Å.

### 3.4. Electrochemical Measurement

LIB cathode electrodes were prepared by mixing S/MPCs (using S/MPC-100 W * 60 s sample, 70 wt.%) with super P (15 wt.%) and polyvinylidene fluoride (15 wt.%). N-methyl-pyrrolidone was added as a binder solvent, and the mixture was stirred magnetically for 10 h. The resulting slurry was coated onto aluminum current collectors and dried at 100 °C under vacuum conditions for 12 h, with a capacity density of approximately 3~4 mAh cm^−2^. Coin cells were then assembled in a dry and inert glove box using the prepared cathodes, Li metal anodes, and Celgard 2400 polypropylene separators. The assembled cells were cycled at different charge/discharge rates over the potential range of 1.3–3.0 V on a BAT-750B cell test instrument (AcuTech Systems Co., Ltd., Taipei, Taiwan). For each test, the specific capacity was calculated on the basis of the amount of sulfur/carbon present. All of the electrochemical tests were performed using 1.0 M LiPF_6_ 1,3-dioxolane/dimethoxyethane (DOL/DML 1:1 by volume) as the electrolyte.

## 4. Conclusions

This study has demonstrated the feasibility for utilizing a simple physical blending technique to transfer bio-pyrolysis oil to value-added MPCs using ZnO as a hard template. Notably, the ZnO template can be easily removed from the synthesized product using hydrochloric acid, and therefore avoids the need for the highly corrosive hydrofluoric acid used in traditional MPC synthesis routes. The characterization results have shown that hierarchical porous carbons with a high specific surface area and an optimum micropore/mesopore ratio can be successfully obtained through carbonization at 900 °C without the need for an additional activation agent. It has further been shown that the pore volume of the MPCs can be easily tuned by adjusting the ZnO/oil ratio. Finally, S/MPC composites have been prepared via a simple microwave sintering process. The coin cell test results have shown that the S/MPC composite material provides a suitable candidate for the cathode electrode in Li-S batteries, with a large reversible capacity about 500 mAh g^−1^ after 70 cycles and a columbic efficiency more than 95%.

## Figures and Tables

**Table 1:** Effect of ZnO/bio-pyrolysis oil ratio on textural properties of MPC samples.

ZnO/Oil Ratio	BET Surface Area/m^2^g^−1^	Micropore Surface Area/m^2^g^−1^	Mesopore Surface Area/m^2^g^−1^	Carbon Yield/%
2	1280	149	1131	29
3	1447	191	1256	24
4	1670	238	1432	18
5	1770	91	1678	13

**Table 2:** Effect of microwave power on textural properties of S/MPC samples.

Condition	Surface Area/m^2^g^−1^	Pore Volume/cm^3^g^−1^	Sulfur Contain/% *
Raw MPC	1447	1.86	-
100 W * 60 s	32.5	0.13	80%
200 W * 60 s	41.9	0.26	73%
300 W * 60 s	42.7	0.39	70%

* The sulfur contain was calculated by weight change before and after microwave treatment as show in Table S1.

## Data Availability

Not applicable.

## Supplementary Materials

The following are available online at https://www.mdpi.com/article/10.3390/ijms23010039/s1.
