# Supplementary material for: Green and Highly-Efficient Microwave Synthesis Route for Sulfur/Carbon Composite for Li-S Battery
Source: Int J Mol Sci. 2021 Dec 21;23(1):39. doi: 10.3390/ijms23010039 (PMC8744887; doi:10.3390/ijms23010039)
Supplement: Supplementary file 1 [file ijms-23-00039-s001.zip › ijms-1510468-supplementary.pdf]

Supporting Information

Green and high-efficient microwave synthesis route for sulfur/carbon composite for Li-S battery

Chun-Han Hsu[a], Cheng-Han Chung[b], Tzu-Hsien Hsieh[c] and Hong-Ping Lin[b]**

a. National Tainan Junior College of Nursing, Tainan 700, Taiwan. b. Department of chemistry, National Cheng Kung University, Tainan 70101, Taiwan. c. Green Technology Research Institute, CPC Corporation, Kaohsiung 81126, Taiwan

*Corresponding author: hplin@mail.ncku.edu.tw; chunhanhsu@gmail.com

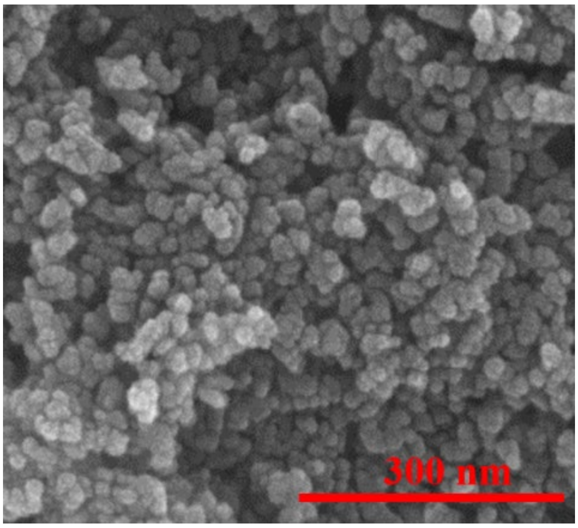

Figure S1. SEM image of ZnO nanoparticle.

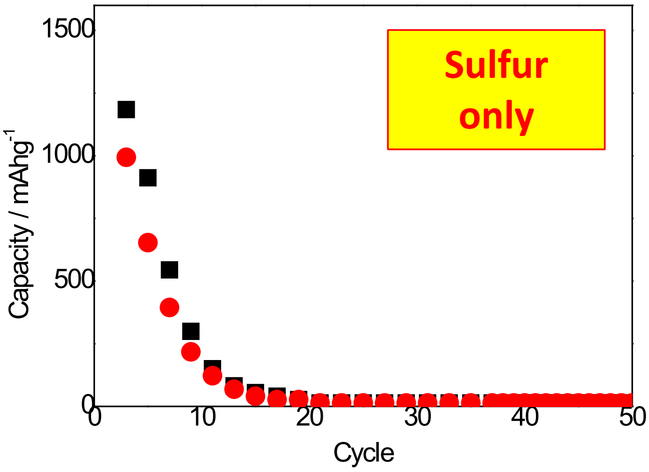

Figure S2. Cyclic performance of sulfur electrode.

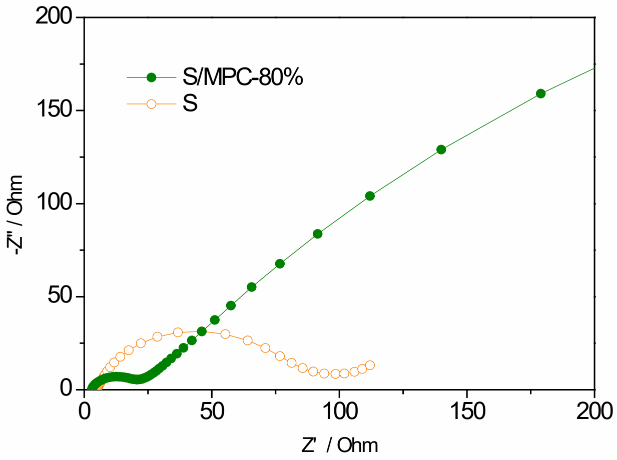

Figure S3. EIS spectra of cell made by S/MPC-80% and sulfur electrode.

Table S1. The weight data of S/MPC samples before and after microwave treatment.

Condition	MPC / g	Sulfur / g	Weight / g (after Microwave treatment)	Sulfur contain/%
Raw MPC	1.0	0	1.0 (*300 W* 60 s*)	0
*100 W * 60 s*	0.2	0.8	1.0	80%
*200 W * 60 s*	0.2	0.8	0.74	73%
*300 W * 60 s*	0.2	0.8	0.67	70%
